# Impact of Surface Polarity on Lipid Assembly under Spatial Confinement

**DOI:** 10.1021/acs.langmuir.2c00636

**Published:** 2022-06-07

**Authors:** Bradley S. Harris, Yuqi Huang, Arpad Karsai, Wan-Chih Su, Pallavi D. Sambre, Atul N. Parikh, Gang-yu Liu, Roland Faller

**Affiliations:** † Department of Chemical Engineering, University of California, Davis, California 95616, United States; ‡ Department of Chemistry, University of California, Davis, California 95616, United States; § Department of Biomedical Engineering, University of California, Davis, California 95616, United States; ∥ Department of Materials Science & Engineering, University of California, Davis, California 95616, United States

## Abstract

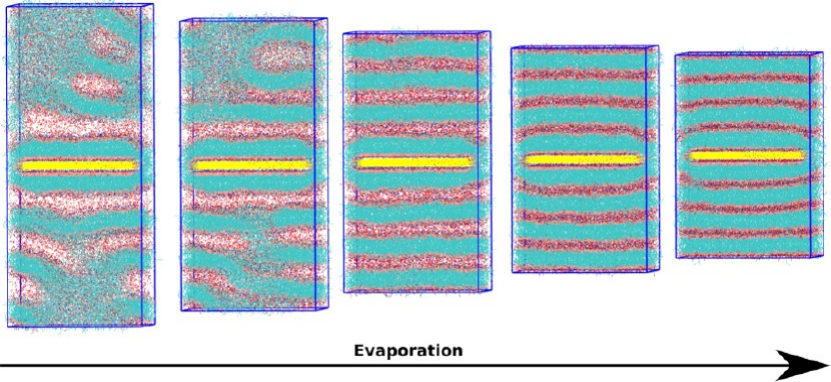

Molecular dynamics (MD) simulations in the MARTINI model are used to study the assembly of 1-palmitoyl-2-oleoylphosphatidylcholine (POPC) molecules under spatial confinement, such as during solvent evaporation from ultrasmall (femtoliter quantity) droplets. The impact of surface polarity on molecular assembly is discussed in detail. To the best of our knowledge, this work represents the first of its kind. Our results reveal that solvent evaporation gives rise to the formation of well-defined stacks of lipid bilayers in a smectic alignment. These smectic mesophases form on both polar and nonpolar surfaces but with a notable distinction. On polar surfaces, the director of the stack is oriented perpendicular to the support surface. By contrast, the stacks orient at an angle on the nonpolar surfaces. The packing of head groups on surfaces and lipid molecular mobility exhibits significant differences as surface polarity changes. The role of glycerol in the assembly and stability is also revealed. The insights revealed from the simulation have a significant impact on additive manufacturing, biomaterials, model membranes, and engineering protocells. For example, POPC assemblies via evaporation of ultrasmall droplets were produced and characterized. The trends compare well with the bilayer stack models. The surface polarity influences the local morphology and structures at the interfaces, which could be rationalized via the molecule–surface interactions observed from simulations.

## 1 Introduction

Construction of nano- and mesostructures of lipid molecules has attracted much interest as these structures serve as precursors for liposomal-based structures upon rehydration to be used in drug delivery and in the production of vaccines.^1−8^ Well-known approaches to build these constructures include “drop-and-dry” approaches^9^ and interruption of phospholipid multilayer constructs that include supported lipid layers (SLBs), Langmuir–Blodgett, Langmuir–Schaefer, and vesicle fusion.^10−12^ While these techniques are experimentally simple and enable the formation of various lipid constructs, the final structures typically consist of a mixture of sizes and compositions. Using the novel concept of “controlled molecular assembly”, our team has developed a new means to form molecular assemblies with designed size and geometry on surfaces.^13,14^ Application of this level of control over lipid molecules would benefit from understanding the complex interplay among lipid–surface interactions, intermolecular interactions among lipid molecules, surface polarity, and the local environment.^15^ This has previously been demonstrated for the assembly of large spherical macromolecules, as well as small nonspherical molecules, such as carbohydrates. The control was based on the delivery of ultrasmall (sub-fL) liquid droplets, which underwent rapid evaporation, forcing the assembly of solute molecules. Applying this approach to lipids faces new challenges, as the intermolecular interactions among POPC, for example, are stronger than that of charged polymer particles or carbohydrates. Here, we perform simulations in conjunction with the experiments to understand the impact and contributions to the lipid assemblies formed under this approach. Due to the significant impact of the initial droplet size and geometry on the evaporation and final assemblies, the surface polarity is anticipated to play a significant role.^13,14^

Molecular dynamics (MD) simulations are one of the most common and powerful techniques used to analyze lipid membrane properties directly.^16^ Despite the ubiquity of MD lipid simulations, there are comparatively few studies investigating the complexity of lipid–surface interactions.^17−28^ All-atom models have been used to accurately describe the confinement of hydration water and surface “lubrication” in SLB systems, but the computational cost to scale up these systems has largely prevented the study of more complex interactions.^19−21^ Coarse-grained (CG) models, on the other hand, have been used to study more complex effects such as lipid vesicle fusion, surface roughness, and inhomogeneity effects.^22−24^ The MARTINI model is extremely popular for lipid bilayer simulations, which provides a level of coarse-graining capable of replicating complex structures while still providing enough molecular detail to compare to experimental data. The MARTINI model has been used previously to study the interactions between lipid bilayers and solid supports and for structural effect studies.^25−27^ Previous studies have especially focused on the difference between the top and bottom leaflets, the effects of topology, and on the vesicle fusion to hydrophobic surfaces.^14−22,26−28^ Most simulations have focused on single aspects of the surface–lipid, surface–solution, and lipid–solution contributions to the behavior of the corresponding experimental SLB systems. In this work, we present MD simulations utilizing the MARTINI CG model, in conjunction with experimental approaches, to study the POPC assembly on hydrophobic and hydrophilic surfaces under various lipid–solvent concentrations. Previous efforts to study lipid–surface interactions at varying hydration levels have focused on hydrophilic surfaces with constant solvent molecules of varying surface thicknesses that change the thickness of the lubricating water layer.^25,29^ To the best of our knowledge, this work represents the first to use MD to study the effect of surface polarity at variable solvent concentrations under spatial confinement. Additionally, we evaluate the impact of evaporation rate by comparing assemblies with and without glycerol to investigate the role glycerol plays in addition to simply slowing down evaporation. This developed method is used to gain insight into the mechanisms of lipid assembly onto surfaces and the corresponding structural evolution during evaporation, as well as the mechanism by which glycerol stabilizes and changes those assembled structures. The insights revealed from the simulation have significant impacts on the construction of nano- and mesoscale lipid structures by design, which will benefit the construction of liposomal-based structures for drug delivery and engineering of vaccines and even protocells.

## 2 Materials and Methods

### 2.1 CG Model Description

We use the coarse-grained MARTINI 3 force field.^30^ This model is based largely on a 4-1 mapping, with four heavy atoms being represented as a single interactive bead, except in the case of some ring-like molecules that have a higher resolution. The model uses four primary CG bead types, C, N, P, and Q, corresponding to nonpolar, intermediately polar, polar, and charged chemical groups, respectively. The nonbonded interactions are described solely by Lennard–Jones potentials between noncharged beads, while charged beads also include Coulombic interactions. Bead sublabels are used to differentiate the degrees of polarity or hydrogen donor/acceptor capabilities. All beads are of the same size except for certain ring structures (S) and certain nucleotides (T). The MARTINI force field has been successfully used to study many biomembranes, proteins, and materials science problems.^31−33^

**Figure 1:**
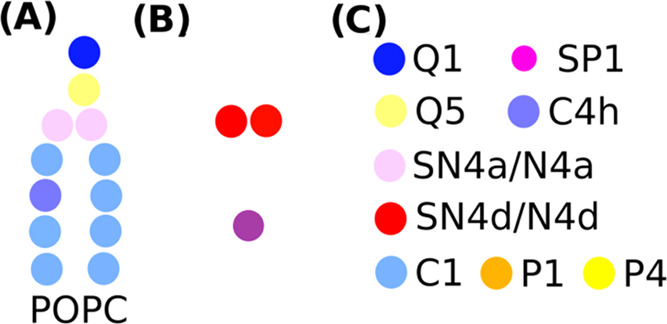
MARTINI three-species representation. (A) Phospholipid POPC; (B) solvent molecules, glycerol (red), and ethanol (magenta); and (C) bead-type key.

The MARTINI representations of the relevant molecular species used here are shown in Figure 1. The phospholipid 1-palmitoyl-2-oleoylphosphatidylcholine (POPC) is the focus of these simulations. POPC is represented by 12 beads, corresponding to a positively charged choline (Q1), a negatively charged phosphate (Q5), two neutrally charged glycerols (SN4a and N4a), and two tails with nonpolar alkane-like beads (C1). The second bead of the unsaturated first chain is represented by a more nonpolar “C4h” bead. Ethanol is the primary solvent molecule, represented with a single bead of type SP1. This corresponds to a smaller bead with a weakly polar interaction strength. The secondary solvent glycerol is represented by the same neutrally charged two-bead system as in POPC, with the modification being a hydrogen donor instead of an acceptor (SN4d and N4d).

### 2.2 Configurations

**Figure 2:**
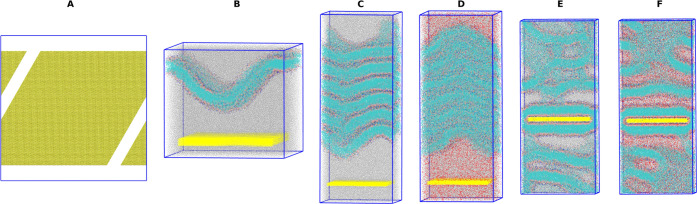
Representative starting configurations for the P4 surface type after 210 ns equilibration before evaporation. (A) Surface structure, (B) one-membrane bilayer start, (C) six-membrane bilayers without glycerol, (D) six-membrane bilayers with glycerol, (E) random start without glycerol, and (F) random start with glycerol.

The initial surface geometry used a regular graphene-like pattern and was built according to the MARTINI tutorial on graphene leaflets.^34^ The surface shape was chosen to avoid using a perfectly flat surface with no edges, as with that level of molecular smoothness, the area per lipid would be artificially fixed. Different surface MARTINI three-bead types were used to model a range of surface hydrophilicities. The chosen bead types were C1, P1, and P4, which represent a nonpolar carbon-like surface, a weakly polar surface, and a strongly polar surface, respectively. Initial POPC lipid configurations were built using the *insane.py* script for bilayer stacks and the *gmx* insert command for random starting configurations.^35^ Starting molecule counts are shown in Table 1, and the initial visualizations are shown in Figure 2. Figure 2 shows the configurations after 210 ns equilibration for the P4 surfaces, Figure 2B corresponds to one bilayer, Figure 2C,D corresponds to six bilayers with and without glycerol, respectively, and Figure 2E,F depicts random starts with and without glycerol, respectively. Following 210 ns equilibration, the configurations that began as membranes (Figure 2B–D) show characteristic signs of thinning due to ethanol but maintain their structure as bilayers at these concentrations.^36^ The random starting configurations in Figure 2E,F show membrane bilayers formed at the surface due to the order imposed by the surface while forming a variety of structures away from the surface. For each surface, the starting configurations studied included a single bilayer, six-bilayer stacks with and without glycerol, and a configuration of randomly placed POPC with the same number of molecules as the six-bilayer stack. The systems that correspond to the initial membrane layers most closely align with the existing methods for SLB formation as a membrane bilayer is introduced to a surface, while the randomly distributed systems most closely align with the experiments carried out in this work, with the introduction of an ethanol solution containing lipids being introduced to a surface.

**Table 1:** Starting Simulation Molecule Counts and Starting Configurations

surface type	# POPC	# EOL	# GLY	# SURF	initial configuration
C1	3042	176 366	0	80 000	one-membrane bilayer
C1	8112	114 092	0	19 600	six-membrane bilayer
C1	8112	97 768	10 000	19 600	six-membrane bilayer
C1	8112	93 745	0	19 600	random
C1	8112	80 813	10 000	19 600	random
P1	3042	176 366	0	80 000	one-membrane bilayer
P1	8112	114 092	0	19 600	six-membrane bilayer
P1	8112	97 768	10 000	19 600	six-membrane bilayer
P1	8112	93 745	0	19 600	random
P1	8112	81 223	10 000	19 600	random
P4	3042	1 76 366	0	80 000	one-membrane bilayer
P4	8112	114 092	0	19 600	six-membrane bilayer
P4	8112	97 768	10 000	19 600	six-membrane bilayer
P4	8112	93 745	0	19 600	random
P4	8112	81 148	10 000	19 600	random

### 2.3 Simulation Protocols

Molecular dynamics (MD) simulations were carried out using Gromacs 2019.1 with the MARTINI 3 force field described above.^30,37^ Following initial configuration generation, a series of minimization steps were performed. Energy minimization was performed twice, before and after solvation with ethanol and in some cases glycerol. The second energy minimization was followed by 300 ps NVT and NAPzT (constant area and constant surface normal pressure) equilibrations at 300 K with a Berendsen thermostat and a 1 atm Berendsen barostat for NAPzT.^38^ Longer equilibration runs were performed in NAPzT with a 300 K Berendsen thermostat and an anisotropic 1 atm Berendsen barostat for 210 ns to ensure the starting structures were sufficiently equilibrated and had no surface interactions. Barostats were applied normal to the surface against a constant surface area to prevent *x*–*y* fluctuations. Evaporation was performed using the methodology described and validated previously to replicate the internal structures formed during evaporation.^13^ Briefly, each evaporation step corresponded to the removal of 1% of the initial ethanol and was simulated for 15 ns per step using the same NAPzT production conditions. The final 1% of solvent was removed at a rate of 0.1% initial ethanol per step. Each evaporation required 110 simulations for full removal, making the total simulated evaporation time of 1.65 μs.

Lipid density profiles were calculated using built-in Gromacs tools, specifically *gmx density*.^37^ All visualizations were performed using *VMD*.^39^ Due to curvature in the lipid membranes during the dehydration process, the area per lipid calculations were performed by Voronoi tessellation using the software *APLVORO*.^40^ These Voronoi tessellations were reconstructed using python and *matplotlib* to show the location of the surfaces. Lipid thicknesses were calculated from density profiles and checked against the Voronoi tessellation results. Velocity profiles were calculated using the *MDAnalysis* streamlines package, and lipid-order parameters were created using the *lipyphilic* python package in python, and plots were generated using matplotlib.^41,42^

### 2.4 Experimental Methods

Lipid microconstructs were fabricated with fluid force microscopy (FluidFM) to compare with the computational analysis. FluidFM takes the use of an independent microfluidic probe on an atomic force microscopy (AFM) stage to produce structures by the extrusion of ink in the fluid state through a nozzle, followed by drying or curing to retain shape.^43^ The experimental method makes use of the rapid evaporation of the minute droplets (842–1162 fL), which can lock the molecules in place. This is in contrast to the conventional method of making lipid bilayer stacks, which relies on self-assembly to fabricate molecules into mesoscale structures and to achieve ordered structures driven by thermodynamics. To the best of our knowledge, the experimental method is the only technology to directly mimic the simulations.

#### 2.4.1 Materials

Glass slides and glass coverslips were purchased from Fisher Scientific (Pittsburgh, PA). Reagents were used without further purification. Glycerol (>99%), sulfuric acid (H_2_SO_4_, 95.0–98.0%), hydrogen peroxide (H_2_O_2_, 30% aqueous solution), ammonium hydroxide (NH_4_OH, 28.0–30.0% aqueous solution), chloroform (99.8%), and toluene (99.8%) were purchased from Sigma-Aldrich (St. Louis, MO). Octadecyltrichlorosilane (OTS) was purchased from Gelest (Morrisville, PA). Ethanol (200 Proof pure ethanol) was purchased from Koptec (King of Prussia, PA). Milli-Q water (MQ water, 18.2 MΩ·cm at 25 °C) was produced by a Milli-Q water purification system (EMD Millipore, Billerica, MA). Nitrogen gas (99.999%) was purchased from Praxair, Inc. (Danbury, CT, King of Prussia, PA). 1-Palmitoyl-2-oleoyl-sn-glycero-3-phosphocholine (POPC) and 1,2-dioleoyl-sn-glycero-3-phosphoethanolamine-*N*-(7-nitro-2-1,3-benzoxadiazol-4-yl) (NBD-PE) were purchased from Avanti Lipids, Inc. (Alabaster, AL).

#### 2.4.2 Preparation of Glass Supports

Three different glass supports were prepared to match the simulations. Coverslips were first cleaned with ethanol and water and then plasma-cleaned for 5 min with a plasma cleaner (PDC-32G, Harrick Plasma, Ithaca, NY). To prepare the OTS/SAM glass coverslips, the glass substrates were first cleaned following established protocols.^10^ Briefly, the coverslips were first cleaned in piranha solution for 1 h and then rinsed with a copious amount of MQ water. Piranha solution was prepared by mixing H_2_SO_4_ and H_2_O_2_ (v/v = 3:1). The coverslips were then soaked in a basic bath, which contains NH_4_OH, H_2_O_2_, and MQ water at a ratio of 5:1:1 (v/v) for 1 h at 70°C. The clean glass coverslips were then rinsed with a copious amount of MQ water and dried in nitrogen gas. The OTS/SAM coverslips were prepared by first immersing the clean coverslips in 5 mM OTS solution in toluene for 3 min. Finally, the modified coverslips were rinsed with toluene and ethanol and dried in nitrogen gas.

#### 2.4.3 Controlled Assembly of Lipid Molecules on Surfaces

The delivery process was carried out using an atomic force microscope (AFM)-based microfluidic delivery platform FluidFM BOT (Cytosurge, Glattbrugg, Switzerland) containing an inverted optical microscope (IX-73, Olympus America, Center Valley, PA). The printing was performed using a FluidFM Nanopipette (Cytosurge, Glattbrugg, Switzerland) with a 300 nm opening. To prepare the lipid solution, POPC was first dissolved in chloroform to make a stock solution of 25 mg·mL^–1^. NBD-PE stock solution was made by dissolving NBD-PE in chloroform at a concentration of 5 mg·mL^–1^. POPC stock solution (39.5 μL) and 2.5 μL NBD-PE solution were mixed together, which was then dried using nitrogen gas to achieve the POPC/NBD-PE mixture. The lipid mixture was then dissolved in the ethanol and glycerol solvent mixture (ethanol/glycerol = 9:1) to 0.033 M.

#### 2.4.4 Characterization of Supported Lipid Constructs

The POPC constructs were left to air-dry and imaged on an AFM (MFP-3D, Oxford Instrument, Santa Barbara, CA). Silicon nitride probes with a resonance frequency of 36 kHz (MSNL-10 E, Bruker, Camarillo, CA) were used to characterize the geometry and size of the printed structures. Image acquisition was done in air under room temperature and ambient condition using tapping mode with 60% damping; the free amplitude was set at 1.10 V, and the scan speed was set to 12.5 μm·s^–1^. Image processing and display were performed using MFP-3D software developed on the Igor Pro 6.20 platform. Particularly, the initial volume of droplets deposited on the surface was estimated from the total number of POPC molecules and the solution concentration. The total number of POPC molecules was calculated based on the feature volume after drying (acquired from MFP-3D developed on the Igor Pro 6.20 platform) and the individual volume (1256 Å^3^) of a POPC molecule.^44^

## 3 Results and Discussion

### 3.1 Lipid Assembly onto Polar and Nonpolar Surfaces during Solvent Evaporation

**Figure 3:**
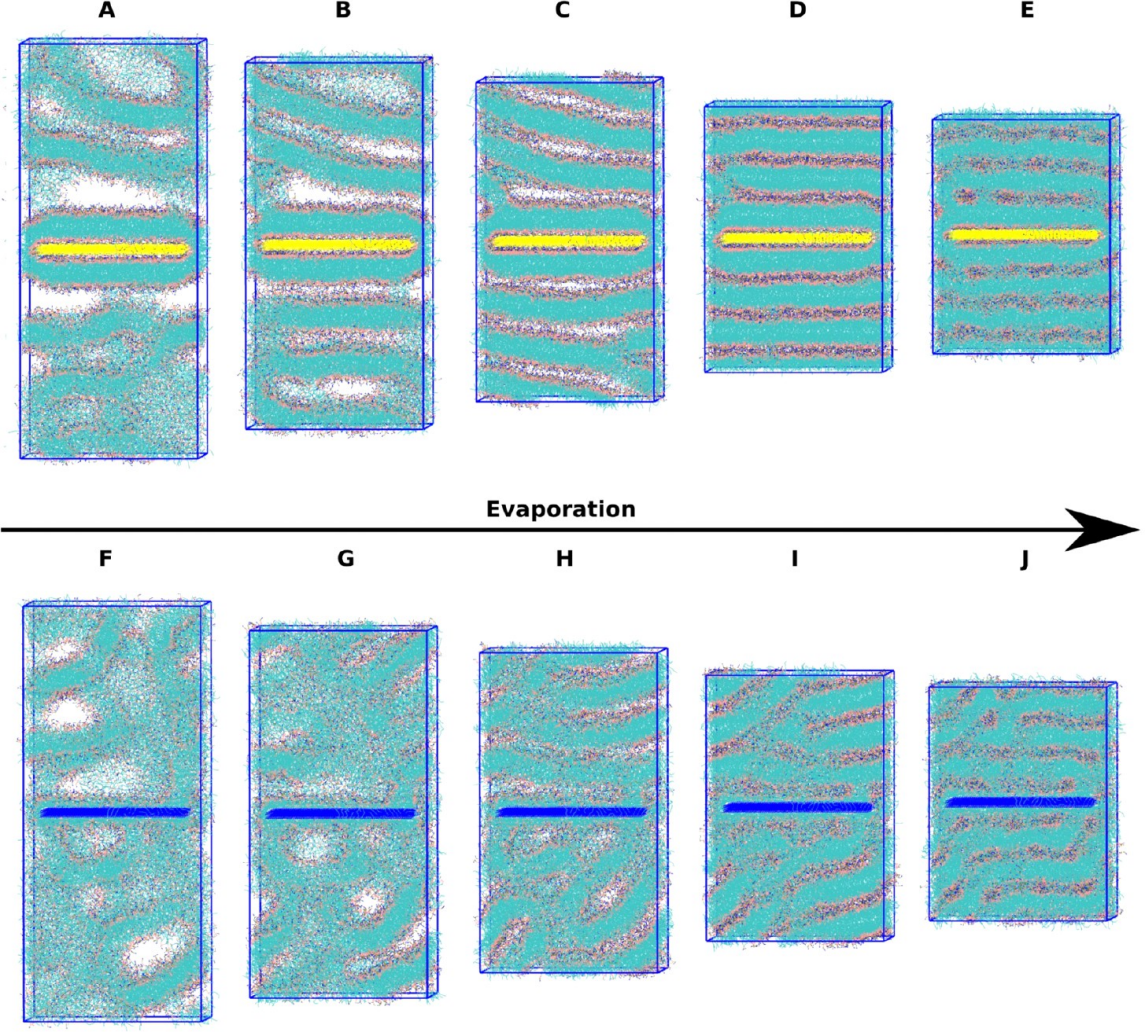
Molecular dynamics snapshots during solvent evaporation on polar and nonpolar surfaces without glycerol, randomly inserted configuration. (A) 100% solvent P4 surface, (B) 75% solvent P4 surface, (C) 50% solvent P4 surface, (D) 20% solvent P4 surface, (E) 0% solvent P4 surface, (F) 100% solvent C1 surface, (G) 75% solvent C1 surface, (H) 50% solvent C1 surface, (I) 20% solvent C1 surface, and (J) 0% solvent C1 surface.

Figure 3 presents the results of computational solvent evaporation on polar and nonpolar surfaces without glycerol. The data shown is from the randomly inserted starting configuration, with Figure 3A–E corresponding to the very polar P4 surface at different solvation levels and Figure 3F–J corresponding to the nonpolar C1 surface at different ethanol solvation levels. Ethanol is removed from visualization for clarity. The initial frames shown in Figure 3A,3F were the result of 210 ns of simulation after initial random lipid insertion. The different surface types result in different modes of attachment, leading to different starting structures. The polar surface has a bilayer formed initially due to a strong polar head group attraction to the polar-terminated surface; the exposed tails then pack together as a typical membrane system would. The nonpolar surface gets covered in a layer of lipid molecules that get attached by their tails, resulting in pockets of head groups and structures that stretch perpendicular to the surface (right side of the image) to minimize solvent interactions with exposed lipid tails. Both systems show a buffer layer of ethanol, visualized as an empty space, between the resulting lipid structures. From 75% remaining initial ethanol to 20% initial remaining ethanol shown in Figure 3B–D, the lipid features away from the surface for the very polar surface have begun to assemble into individual lipid bilayers that are gradually forced together during the dehydration process. At 20% initial solvation, the lipid bilayers from the surface appear to be very uniform, with a thin layer of ethanol between them. The relative ratio of lipids to ethanol is 1 POPC: 2.81 ethanol at this point. From 20 to 0% of initial solvation, the thin layer of ethanol is removed, and the layers begin to interpenetrate and form less ordered subdomains in the bulk. Similarly, the nonpolar C1 surface shown in Figure 3F–J shows a compacting of the initially formed lipid features into more uniform lipid bilayers, with the most ordered layers shown in Figure 3I, and the interpenetration and mixing at a 0% initial solvation (Figure 3J). The nonpolar surface takes longer to form uniform layers, possibly due to a much less ordered initial configuration owing to the tail–surface interaction preference or the resulting lower surface tension. Movies showing the full evaporation and surface attachment process for every system are available in the SI.

While the random starting configurations are most directly comparable to our experimental deposition process, the stacked membrane bilayers are comparable to Langmuir–Blodget or Langmuir–Shafer deposition methods for SLBs. When analyzing the stacked membrane bilayer starting configurations (Figures S1 and S2), we find that the process of lipid adsorption to a surface during the dehydration process is comparable to those observed previously in simulations under constant hydration conditions.^4,20−22,24−28^ This similarity is clearly seen by the presence of the lubrication effect or a hydration layer forming between the surface and the lipids, the gradual sliding effect of the lipids onto the surface, and the inversion of the lipid membrane on the nonpolar surface. In our experimental deposition process, ethanol is used as the primary solvent instead of water to avoid aggregation of lipid, resulting in the blockage of the tip, as well as for its volatility given the evaporation-driven nature of the assembly. Ethanol has been shown computationally to result in the thinning of lipid membranes, the dissolution of lipid vesicles at high concentrations, a decrease in the distance in lipid vesicles, and the disruption of membrane bilayers.^36,44−47^ For POPC specifically, it has been shown that ethanol has high permeability and aggregates at the hydrophilic/phobic interface.^44,45^ Shobhna and Kashyap^47^ show that at significantly higher ethanol-to-lipid ratio vesicles still retain structures. When analyzing snapshots of solvent permeability (Figures S3–S5), we observe evidence consistent with these trends of thinning, pore formation, and high permeability across POPC bilayers, as well as ethanol aggregation at the hydrophilic/phobic interface.

### 3.2 Effect of Glycerol on Lipid Assembly

**Figure 4:**
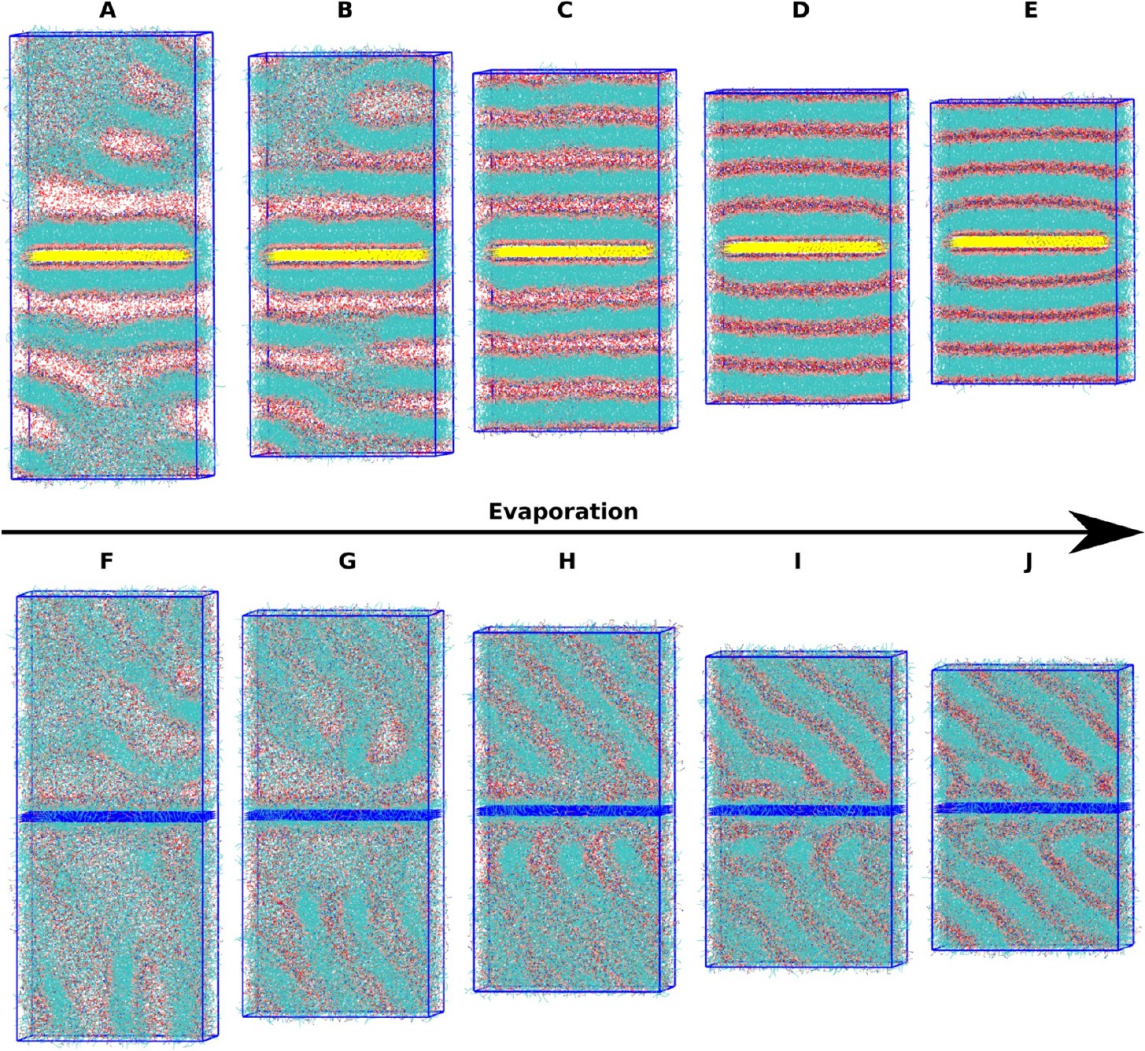
Molecular dynamics snapshots for solvent evaporation on polar and nonpolar surfaces with glycerol, six bilayers starting configuration. (A) 100% solvent P4 surface, (B) 75% solvent P4 surface, (C) 50% solvent P4 surface, (D) 20% solvent P4 surface, (E) 0% solvent P4 surface, (F) 100% solvent C1 surface, (G) 75% solvent C1 surface, (H) 50% solvent C1 surface, (I) 20% solvent C1 surface, and (J) 0% solvent C1 surface.

Experimentally, glycerol is known to stabilize and preserve lipid solutions.^48^ Computationally, previous atomistic MD studies have shown that glycerol forms a cross-linked network on the surface of lipid membranes contributing to this stabilizing effect.^48^ In our experiments, glycerol is added to slow the evaporation rates to improve the control of the liquid environment being deposited. To investigate the effect this glycerol addition plays on the formation of lipid structures, we added 10 000 glycerol molecules to the simulations shown in Figure 3. Snapshots are shown in Figure 4 at the same ethanol solvation levels shown previously. Glycerol is not removed during the simulated evaporation due to its much lower volatility compared to ethanol and to study the effect of the remaining glycerol on the final structures. Figure 4A–E corresponds to the highly polar P4 surface, and Figure 4F–J corresponds to the nonpolar C1 surface. Ethanol is once again removed from the visualization for clarity. The first half of ethanol removal is very similar for the strongly polar surface when compared to the system without glycerol (cf. Figure 3A–C). The final 50% of ethanol removal shows a deviation in the formation of final structures. There is clearly more separation between the layers attached directly to the surface and the bulk in Figure 4D,E. Additionally, glycerol shows a stabilizing effect by adding a buffer layer between POPC layers resulting in more uniform bilayer stacks beginning in Figure 4C and continuing through Figure 4E. Bilayer dissipation due to increased lipid interaction is not present close to the surface as a result of this effect. Similar trends are observed for the nonpolar C1 surface. Initially, the surface shows lipid tail–surface interactions with pockets of head groups and a less ordered structure above the surface buffered by solvent. At 75% of initial ethanol, the glycerol is beginning to stabilize the lipid structures away from the surface, with a clear separation beginning to form between layers. At 50% initial ethanol solvation, the more ordered layers from the solution have begun to form layers diagonally from the surface, likely between the pockets of head groups to minimize the tail–solvent exposure. At 20% ethanol solvation, the glycerol has begun to separate the layers above the surface in addition to the layer closest to the surface. Finally, at 0% ethanol solvation, the glycerol has aided the formation of bilayers across the entire system and prevented interpenetration, with glycerol isolating the layers and the pockets of head groups attached to the surface. The movies for these systems are also available in the SI.

Glycerol clearly provides a larger buffer layer compared to the ethanol layer. This larger layer is observed at both the surface and the layers between the lipid layers. These glycerol buffer layers are clearly visible in Figure 4. For the systems that started as stacked bilayers, the penetration depth of the glycerol layers extends several layers but not to the final layer (Figure S2). This effect is comparable to the effects observed with the introduction of cryosolvents on lipid structures, whereby a cross-linked network forms on the surface of the head groups.^48^ Zoomed-in snapshots of these glycerol interactions with membrane bilayers, as well as competing ethanol effects, are shown in the SI (Figures S3–S5). Our simulations show that these cross-linked networks isolate and stabilize individual bilayers in dehydrated structures.

### 3.3 Quantification of Resulting Lipid Structures

**Figure 5:**
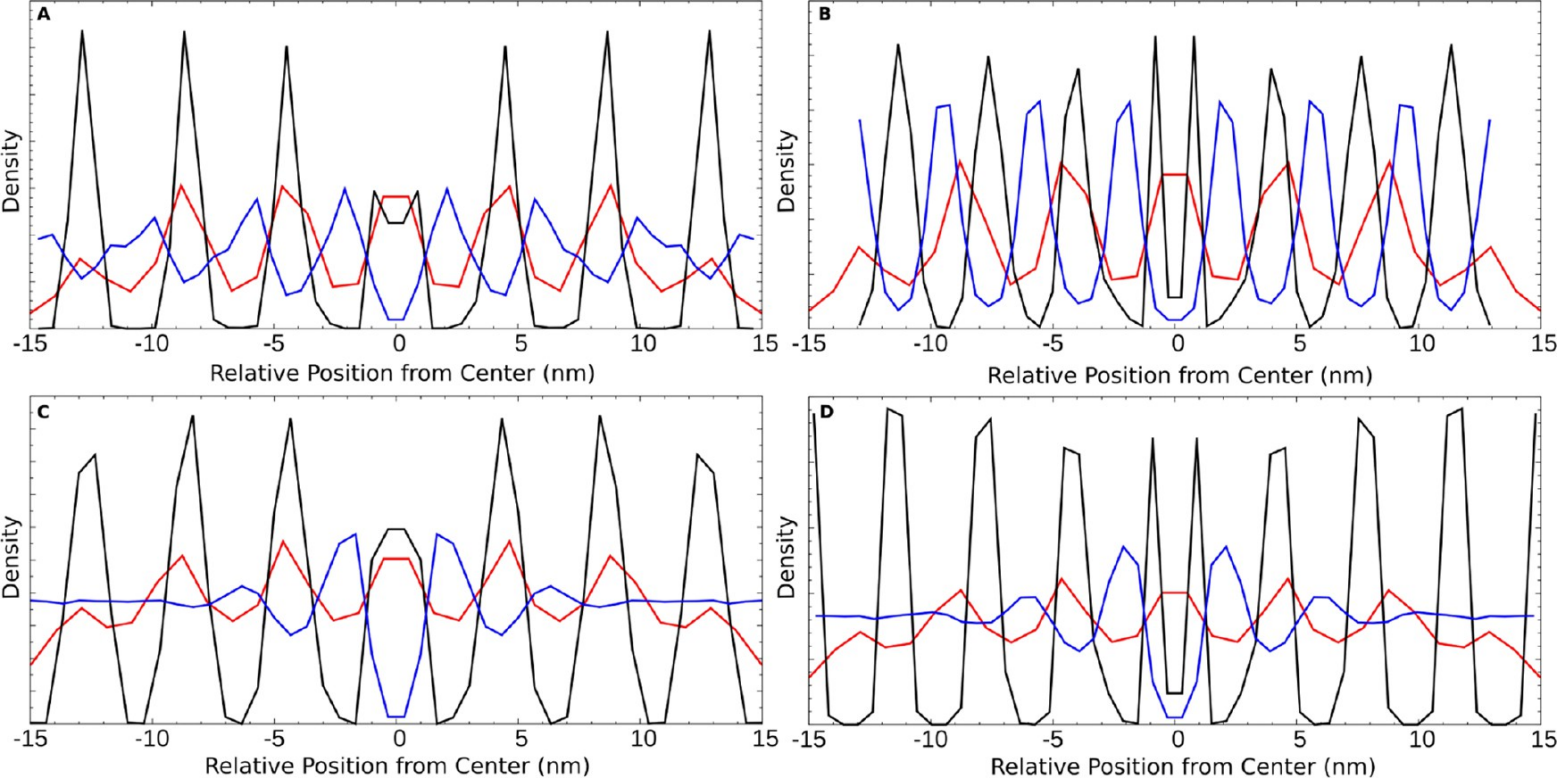
Lipid density profiles in the *z* direction (bilayer normal) and centered around the center of mass of the phosphate head group beads. (A) No glycerol 20% of initial ethanol from random start. (B) No glycerol 0% of initial ethanol from random. (C) 20% of initial ethanol with glycerol from random start. (D) 0% of initial ethanol with glycerol from random start. The red line corresponds to the control bilayer stack with or without glycerol, black lines correspond to the very polar P4 surface, and blue lines correspond to the nonpolar C1 surface.

To quantify these observations, lipid density profiles were calculated (Figure 5). Figure 5A,B corresponds to the systems without glycerol and random start at 20 and 0% solvation, respectively, and Figure 5C,D corresponds to the systems with glycerol and random start at 20 and 0% solvation, respectively. Red lines correspond to the density profile of the stacked membrane away from the surface at full solvation without and with glycerol as a control. Black lines correspond to the very polar P4 surface, and blue lines correspond to the nonpolar C1 surface. Figure 5A shows that lipid stacks formed on the surface closely align with those freely floating in solution. The C1 surface shows maximum peaks with an offset from the center due to the attachment of tails to the surface. The maxima of these peaks appear to roughly correspond to a similar thickness as the control group, with a similar offset for each peak. The nonpolar peaks, however, are broader, suggesting the presence of lipid head groups in locations not corresponding to perfect bilayer stacks. Compared to the visualization in Figure 3I, these likely correspond to the bilayer stacks not forming at an angle to the surface rather than parallel. The fully dried structures shown in Figure 5B demonstrate a thinning of the membrane compared to the control profile. The peaks for both surfaces become more uniform, with the same tail–surface-related offset for the C1 surface and slightly broader peaks for the C1 surface related to the slight angle of the structure. The effect of the addition of glycerol is shown in Figure 5C,D. For the polar surface, the overall trends are similar, almost matching the control in Figure 5C and thinning in Figure 5D. There appears to be a general broadening of the peaks for all systems in the presence of glycerol and a slight offset from the control group in 5Figure 5C for the very polar surface. The assembled structures on the C1 surface show a dramatically different profile in the presence of glycerol. In Figure 5C,D there are two distinct peaks on either side of the surface (*z* = 0), with the same offset corresponding to the tail–surface attachment. Beyond the two peaks, however, there is an even distribution of phosphate beads away from the surface, making it difficult to distinguish between membrane bilayers in the *z* direction. Comparison to the visualizations in Figure 4I,J suggests that this is due to the angle the membrane bilayers have formed on the surface. Density profiles for the starting bilayer stack and other configurations that are not shown here are available in the Supplementary Information (Figure S6).

Thickness values from Figure 5 were calculated and are shown in Table 2 along with lateral diffusion coefficients for these states. Table 2 clearly shows that the membranes at 20% remaining initial ethanol align with the initial membrane away from the surface for both membrane thickness and lateral diffusion behavior. Lateral diffusion coefficients obtained using MARTINI force fields are known to be higher than those obtained experimentally, especially in the case of nonadjusted simulation time. Our lateral diffusion coefficients are in a similar range to those observed previously in the literature when MARTINI has been used.^31,49^ As these structures are further dehydrated, the membranes break up resulting in interdigitation, as shown previously; this results in a decreased membrane head–head thickness. Interestingly, this effect is shown to also occur in the presence of glycerol. Lateral diffusion slows down with dehydration, as the membranes become more locked in and with less fluid. The presence of glycerol increases the fluidity of these membranes, resulting in a less dramatic slowdown in lateral diffusion.

**Table 2:** Thickness and Lateral Diffusion Coefficients from Random Starting Configurations with and without Glycerol

		C1		C1 (glycerol)		P4		P4 (glycerol)	
parameter	initial	20% EtOH	0% EtOH	20% EtOH	0% EtOH	20% EtOH	0% EtOH	20% EtOH	0% EtOH
thickness (nm)	4.14	4.20	3.69	4.65	3.63	4.18	3.70	4.07	3.47
*D* (10^–5^ cm s^–1^)	0.037	0.034	0.013	0.034	0.021	0.037	0.013	0.049	0.031

### 3.4 Mechanisms Associated with Lipid Assembly during Dehydration

To study the mechanistic behavior of the POPC assembly during simulated evaporation, the potential energy, surface tension, and lipid tail-order parameter were evaluated. The potential energies as a function of the percentage of remaining ethanol are shown in SI Figure S7. Figures 4A and S4B highlight the differences due to starting configuration for simulations without and with glycerol, respectively. All surfaces and configurations are shown in the SI (Figures S7–S9). For starting configuration comparisons in Figure S7A,B, the systems that start as six-stacked bilayers have lower initial potential energy before the final structures converge in the final 20% of ethanol solvation. This is expected as the membrane bilayers will be in a more energetically favorable state compared to the distributed mixture. The convergence of all systems suggests that the starting configuration affects the mechanism associated with surface attachment but not the energetic favorability of the final structure when all ethanol is removed. At slightly higher ethanol hydration, there remains a small difference in potential energy caused by the starting configuration, likely related to the distribution of solvent in the final structures.

These plots clearly show that glycerol reduced the potential energy values when compared to their counterparts without, again supporting the notion that glycerol stabilizes the lipid assemblies. Points corresponding to the C1 surface with the bilayer stack starting configuration show a slight dip around 82% initial remaining ethanol. For nonpolar surfaces, it is known that lipid micelles and membranes go through an inversion when attaching to the surface.^20^ Visual inspection from the simulation videos of evaporation included in the Supporting Information reveals that this dip likely corresponds to this inversion and surface attachment, resulting in a slight decrease in potential energy. The presence of glycerol appears to mask the changes in potential energy during the evaporation process, with no visible dips, and what appears to be a constantly increasing slope compared to the changing plots without glycerol. For all systems, the nonpolar C1 surface has higher potential energy values as compared to the highly polar surface, demonstrating less energetic favorability.

**Figure 6:**
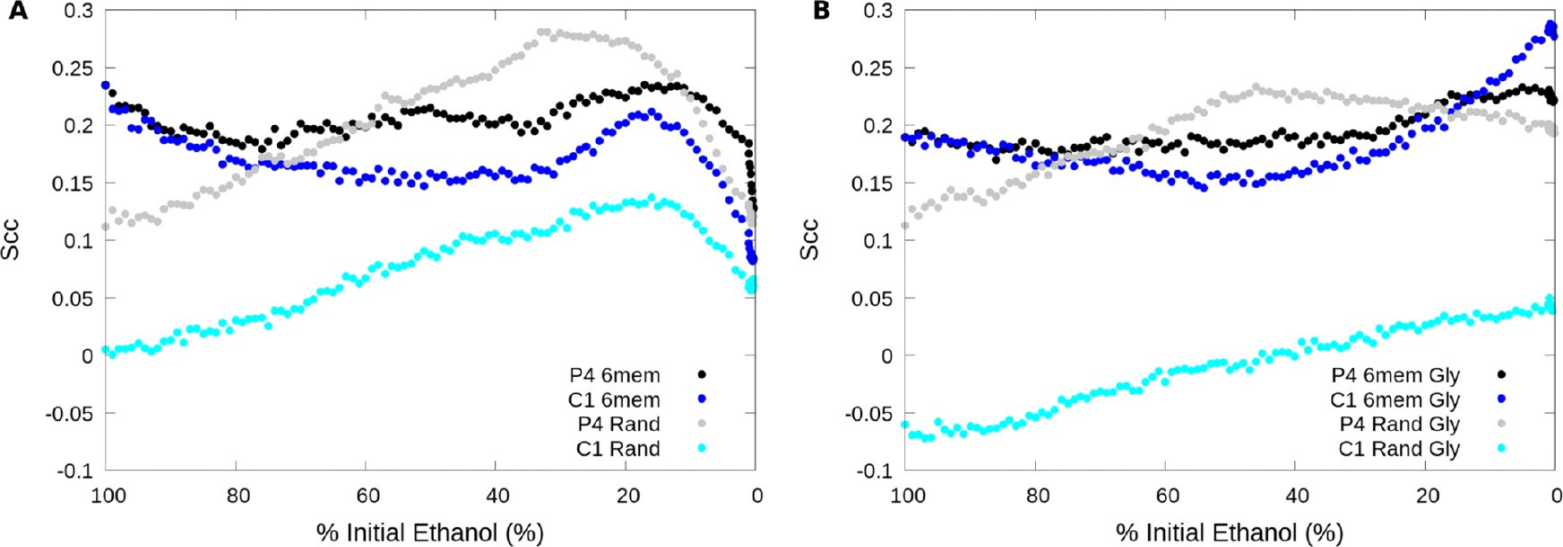
Coarse-grained lipid tail-order parameter as a function of the % of remaining initial ethanol. (A) Bilayer stack vs random without glycerol. (B) Bilayer stack vs random with glycerol.

To further investigate the contributions of starting configuration and glycerol on the evaporation mechanism, the surface tensions as a function of ethanol concentrations are shown in Figure S8. Figure S7A,B corresponds to the same comparisons as in Figure S7, starting configurations without and with glycerol. Beginning with the starting configuration comparison, clearly the polar surfaces have a higher surface tension for all states but the stacked bilayer starting configuration with glycerol. This is likely due to the differences in the starting location of the glycerol. The random starting configuration was more evenly distributed, while the six bilayers trapped more glycerol at the surface and were not able to diffuse and distribute evenly across the structure, resulting in differences in surface tension as one moves away from the surface. The presence of glycerol seemingly results in more structured membranes in the random start when the glycerol is more evenly dispersed in the solution. The nonpolar surfaces all converged with low surface tension for all configurations and solutions. Nonpolar stacked bilayer without glycerol shows an increase in surface tension beginning around 82% remaining initial ethanol, providing further evidence that the transition was related to the initial process of lipid inversion onto the surface, eventually transitioning back to the final values for the random configuration. Finally, the coarse-grained lipid tail-order parameter was evaluated for the same systems and is shown in Figure 6. The coarse-grained lipid-order parameter is calculated, as shown in eq 1, with θ representing the angle between the corresponding bond and the bilayer normal. Values shown correspond to the average between both POPC tails, averaged for all POPC molecules over the 15 ns simulation at each ethanol concentration
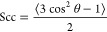
1The systems that start as bilayers show a high tail order due to having an ordered bilayer starting structure. This structure breaks up during evaporation resulting in a drop of lipid order between 90 and 40% solvation. Below 40% solvation, the structure gains order until peaking around 20% solvation. After 20% solvation, the structure starts to disintegrate as the tails can interact with the layers that were separated by ethanol. The randomly generated structures show a linear increase in tail-order parameter, peaking around 35% and then decreasing without the presence of glycerol and plateauing in its presence. The polar surfaces in the random starting configurations show a higher tail-order parameter compared to the nonpolar surface due to the presence of an ordered bilayer at the surface owing to the strong head group interactions. This suggests that the presence of ethanol stabilizes the structures that are present and prevents lipid interdigitation. The addition of glycerol appears to stabilize the layers that are formed on the surface as or more strongly than the thin ethanol layer remaining at 20% for all configurations.

Lipid assembly from the random starting configuration on the C1 surface shows a lower tail-order parameter during the entire process with and without glycerol. Correlating this with the visualizations in Figures 3F–J and 4,3F–J suggests the difference in the final states as being related to the off-parallel angle the structures eventually form at. At full solvation, the system is expected to be less ordered given the lack of a bilayer formed on the surface similar to that formed on the polar surface. Clearly, the drying process causes the lipids above the surface to be less ordered for the C1 surface compared to those for the P4 surface. This suggests that the local structure at the surface affects the assembly and lipid behavior of the surrounding region. Taking all of this together suggests that the presence of glycerol strengthens the formation and stability of bilayers due to the cross-linked network formed between the bilayers, especially as the system is dehydrated. This stabilizing effect suggests that glycerol could act like a sugar would in the water replacement hypothesis.^29^ On nonpolar surfaces, the glycerol appears to stabilize the angle the lipid structure is formed at.

### 3.5 Effects of Surface Polarity on the Assembly Lipid Molecules

**Figure 7:**
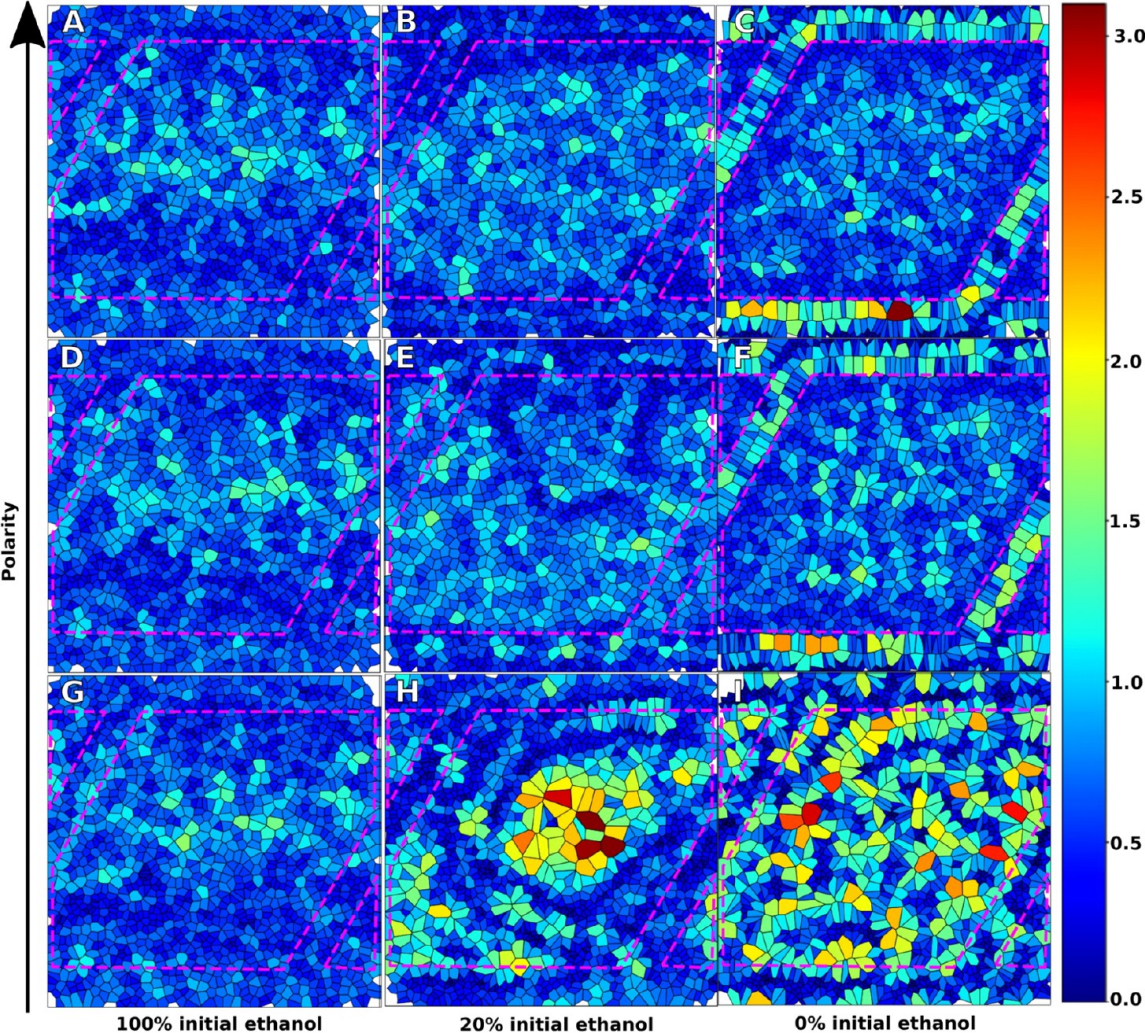
Voronoi tessellations of the area per lipid. Cells correspond to PO4 beads in the lipid head group normalized to a two-dimensional (2D) plane, generated from one-bilayer starting configuration. (A–C) P4 surface at 100, 20, and 0% ethanol solvation, respectively. (D–F) P1 surface at 100, 20, and 0% ethanol solvation, respectively. (G–I) C1 surface at 100, 20, and 0% ethanol solvation, respectively. Broken pink lines indicate the shape of the surface. The scale bar corresponds to the area (Å^2^) per lipid.

Due to the curvature and continuous breakup and re-formation of lipid membranes during simulated drying, area per lipid calculations using standard approaches using leaflet tracking were difficult. To enable the calculation of area per lipid, Voronoi tessellations were performed on the one-bilayer starting structures for each surface at 100, 20, and 0% solvation (cf. Figure 7). Figure 7A–C corresponds to the P4 surface, Figure 7E,F corresponds to the P1 surface, and Figure 7G–I corresponds to the C1 surface. The starting structures at 100% solvation look similar and show a band of higher area per lipid due to the starting curvature in the lipid membrane, as seen in Figure 2A. This membrane then gradually attaches to the surface and forms pockets that resemble liposomes. The remnant of one of these structures can be seen in Figure 7E in the center of the surface. The polar surfaces behave very similarly, though at 20% solvation the presence of this ring has dissipated for the strongly polar surface, suggesting that the interaction strength affects the speed at which the lipids adhere to the surface. At 0% solvation, the polar surfaces show multiple phases, a closely packed structure in the edges and gaps between the surface, and the layer formed on top of the structure. This contrasts with the behavior of the nonpolar C1 surface that adheres to the surface through tail groups, forming pockets of head groups with extremely high area per lipid, as shown in Figure 7H. At 0% ethanol solvation, the nonpolar surface exhibits a very different local phase as compared to the polar surfaces. This suggests that the head groups packing to the surface result in a highly predictable layer with tight packing in the edges, while the nonpolar surface results in islands of head groups, with some having high area available and some with low area available. Interestingly, this pattern does not follow the surface boundary as closely as in the case of the polar surfaces.

**Figure 8:**
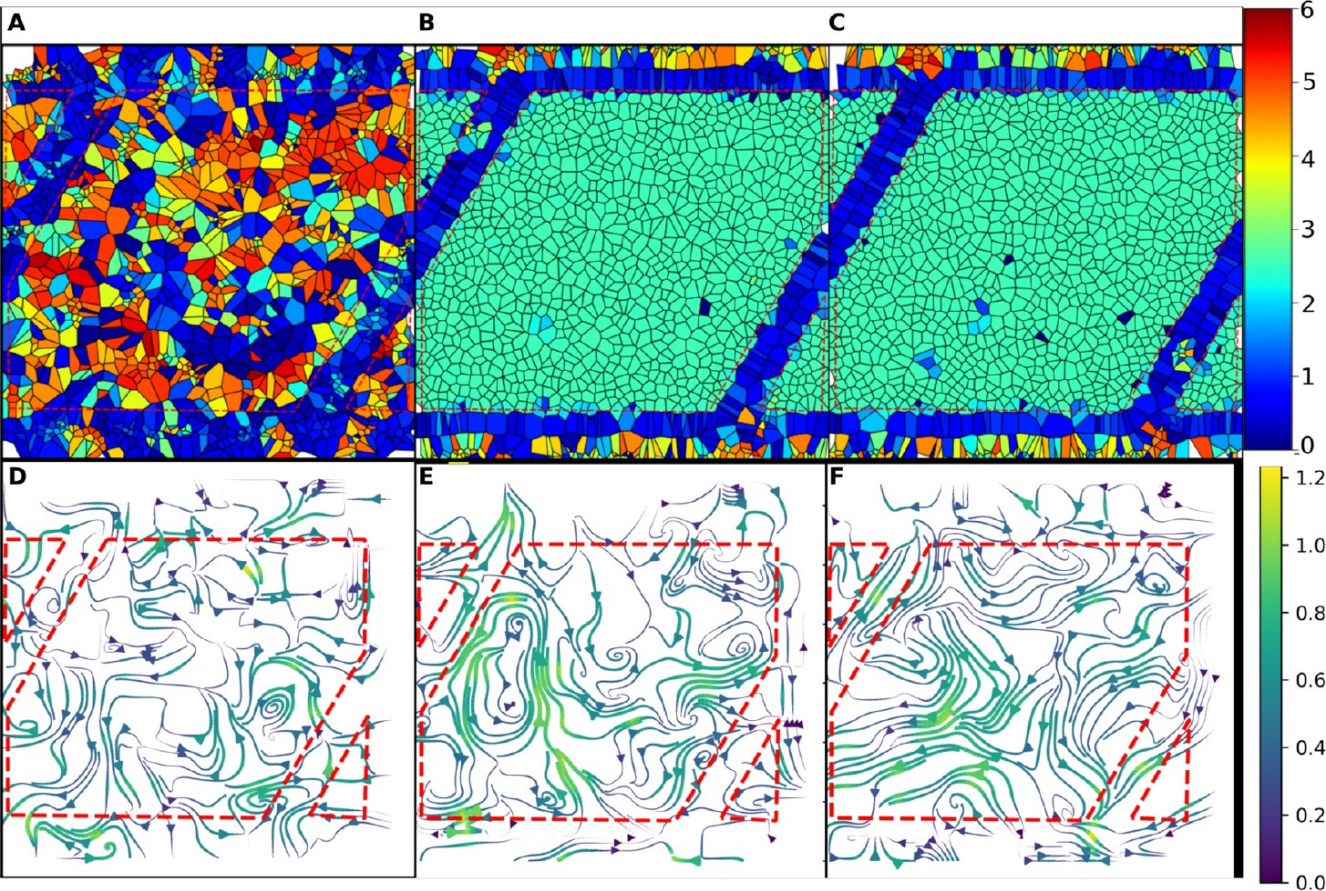
Lipid thickness and surface velocity plots. Lipid thickness applied to Voronoi tessellation plots. Velocity streamlines calculated at the surface for the final 15 ns of simulation time. All structures at 0% ethanol solvation from one-bilayer starting configuration. (A) C1 surface thickness, (B) P1 surface thickness, (C) P4 surface thickness, (D) C1 velocity profile, (E) P1 velocity profile, and (F) P4 velocity profile. (A–C) Scale bar is thickness in nanometer. Panels (D–F) is the velocity in Å/1.5 ns based on frame resolution.

These Voronoi tessellations were also used to calculate and visualize the thickness of the layer directly above the surface. To inspect the mobility of these lipids, the streamline function in MDAnalysis was used to calculate the velocities of the lipid head groups along the surface.^39^ The velocity in this case is the distance the phosphate groups travel in the lateral *x*–*y* dimensions in angstroms at each frame. The velocities were calculated for the 15 ns trajectory reduced to 10 frames, resulting in a denominator of 1.5 ns. The results of these calculations are shown in Figure 8. Figure 8A,D corresponds to the nonpolar C1 surface, Figure 8B,E corresponds to the polar P1 surface, and Figure 8C,F corresponds to the strongly polar P4 surface. The homogeneity of the polar surfaces is clearly demonstrated by the thickness profiles in Figure 8B,C, while the heterogeneity of the nonpolar surface in Figure 8A due to the distinct lipid domains is clear. Interestingly, the mobility of the polar head groups is much higher for the polar surfaces in Figure 8E,F compared to the nonpolar surface in Figure 8D. This is manifested in the number of streamlines as well as the sustained high velocity levels. The direction of the head group motion associated with the streamlines suggests mobility across the entire leaflet on the surface, while the head groups on the nonpolar surface get confined to locally ordered subdomains.

Surface functionality clearly plays a role in the resulting structures from this dehydration process. The area per lipid and Voronoi tessellation analysis reveal that the surface type affects the homogeneity of the layers that are attached to the surface. Polar surfaces result in an attachment of the head groups to the surface, with stronger polarity resulting in a stronger attachment. The nonpolar surface type results in a more distributed attachment of the tails, which forms islands of the head groups that appear less structured on the Voronoi tessellations. Velocity analysis of these structures reveals a difference in the dynamics of these head groups that are attached to the surface. On a polar surface, the lipids are still mobile and capable of moving around on the surface with a velocity pattern that traverses the entire surface. The nonpolar surface results in much less surface velocity, with motion confined, likely due to the increased number of interactions per molecule (tails to surface) resulting in stronger binding of the tail groups to the surface.

### 3.6 POPC Assemblies upon Solvent Evaporation from Ultrasmall Droplets

**Figure 9:**
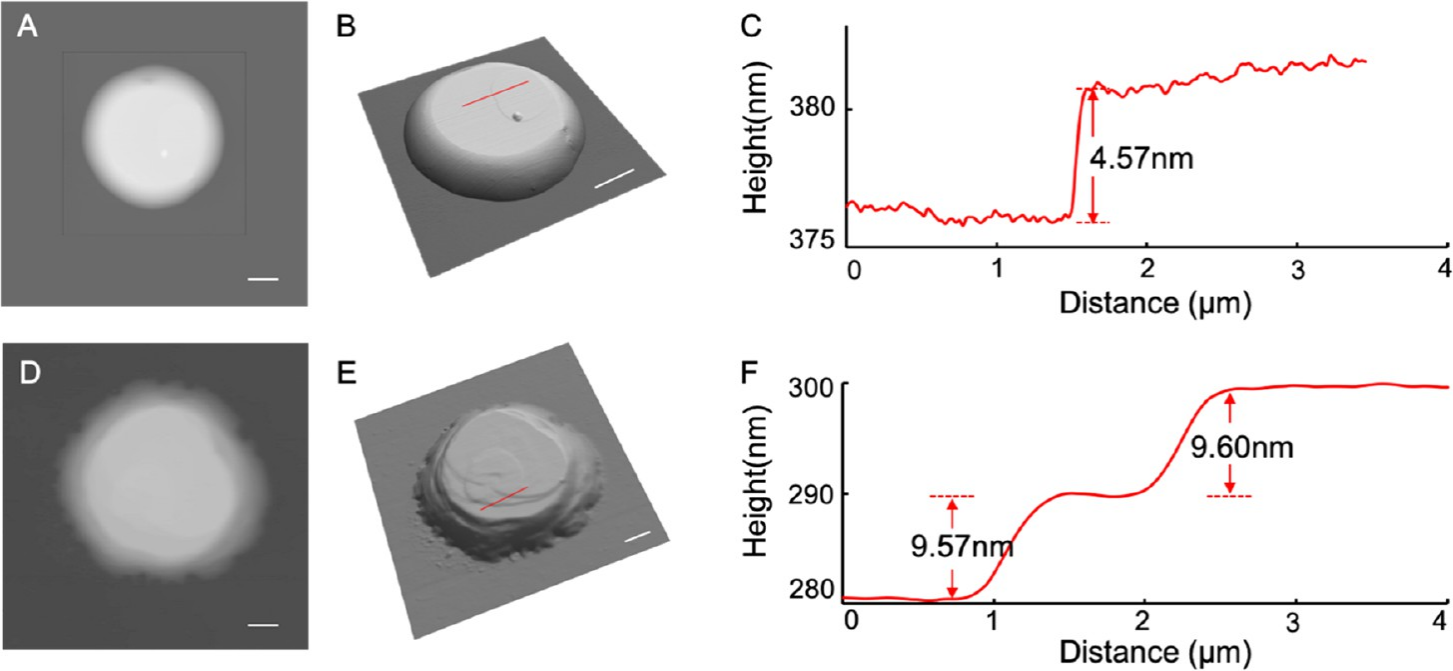
(A) AFM topographic image of a POPC assembly on a plasma-cleaned glass slide. (B) A 3D display of panel (A). (C) A cursor profile as indicated in panel (B). (D) An AFM topographic image of a POPC construct on an OTS SAM/glass surface. (E) A 3D display of panel (D). (F) A cursor profile as indicated in panel (E). All scale bars are 2 μm.

We performed experiments to compare with the computational analysis. In the case of the polar surface, plasma-cleaned glass slides were used as supports. The water contact angle on these surfaces was near 0°. Following the delivery of 0.033 M POPC solution and allowing it to dry for 3 days under ambient conditions, the resulting assemblies were imaged using AFM. The volume delivered was estimated and calculated using the method described above to be 842 fL. A representative POPC structure formed under the delivery condition of a 10 mbar extrusion pressure and a 10 ms contact time is shown in Figure 9A, with the overall geometry resembling a truncated cap. The base and top diameters measure 9.5 ± 0.1 and 6.3 ± 0.2 μm, respectively, with a height of 382 nm. Terraces with steps were clearly observed on top of the assembly, as seen in the 3D display (Figure 9B). The height profiles showed a layer thickness of 4.57 ± 0.56 nm (Figure 9B,C), which is consistent with an interbilayer distance (4.51 nm) of POPC bilayer stacks prepared on silicon wafers using the “rock-and-roll” method.^50^ Thus, we infer that the assemblies prepared in our approach consist primarily of stacks of POPC bilayers.

In the case of the nonpolar surface, octadecyltrichlorosilane (OTS) SAMs were formed on clean glass slides. The water contact angle on these surfaces measured 39.0 ± 0.7°. Following identical delivery conditions (10 mbar extrusion pressure and 10 ms contact time) as that used in Figure 9A, the POPC solution with an estimated volume of 1162 fL was delivered onto the surface and allowed to dry for 5 days. The assemblies of POPC were characterized by AFM. A representative structure of these assemblies is shown in Figure 9D, where the geometry can be described as a truncated cap with rough edges. The base boundary exhibited a deviation from a smooth circle, and the side edges also appeared roughened. The lateral dimensions of the base and top measure 14.0 ± 0.6 and 8.5 ± 0.2 μm, respectively, with a height of 306 nm. The top surface appears rougher than that in Figure 9A,B, as clearly visible in the three-dimensional (3D) display in Figure 9E. The two step heights, as indicated in Figure 9E,F, were measured to be 9.60 ± 0.26 nm (top) and 9.57 ± 0.22 nm (bottom), respectively. The heights mathematically equal 2 × (4.80 ± 0.13) and 2 × (4.78 ± 0.11) nm, respectively, suggesting that each step had two bilayers. These interlayer distances (4.80 and 4.78 nm) appeared larger than that in Figure 9A (4.57 ± 0.56 nm) and the known bilayer thickness reported previously (4.51 nm).^50^ A very tempting explanation for this fact could be the simulation results shown in Figure 4I, which suggests that the lipids stack at an angle from the surface instead of parallel. We must note that another possibility is the presence of solvent molecules, such as glycerol molecules trapped in the interlayer space. An additional structural characteristic that differs Figure 9D from Figure 9A is the deviation from the geometry of a truncated cap, e.g., (a) 17 lipid tendrils at the base and (b) rough step edges along the side. The geometry of these lipid tendrils is lamellipodia-like. The relatively sharp podia measure 0.9–1.1 μm wide at the base, extruding outward by 0.5–0.7 μm. The heights at the bases measure 13–25 nm. The others are 1.7–4.4 μm wide at the bases, extruding 0.6–1.3 μm outwards. The heights at the bases measure 33–74 nm. The formation of these podia is consistent with strong POPC tail groups—OTS interactions, which reduced the mobility of lipid and formed nucleation sites during drying, as shown in Figure 8, leading to podia features at the boundaries. These base layers subsequently impacted the packing of subsequent POPC layers.

## 4 Conclusions

A collection of molecular dynamics simulations utilizing the MARTINI CG model were used to study the interplay between POPC and hydrophobic and hydrophilic surfaces as a function of the relative lipid–solvent concentration. The simulations extend beyond previous efforts in studying lipid–surface behavior that focused mostly on hydrophilic surfaces and a narrow-fixed range of concentrations. To the best of our knowledge, this work is the first to use MD to study the assembly of lipids during solvent evaporation from ultrasmall droplets and to study the effect of surface polarity and at a wide and continuous range of concentrations. Additionally, the significant roles of glycerol are revealed in the assembly of lipid molecules during the drying process. MD simulations demonstrate that the surface polarity affects the local structure of lipid assemblies, such as increased structural homogeneity with increasing polarity. The different surface polarities also dictate whether the dominant interactions shall be head–surface or tail–surface, resulting in uniform parallel bilayer stacks in the cases of strong head–surface interactions and angled bilayer stacks in the cases of strong tail–surface interactions. The nonpolar surfaces exhibit stronger interactions with the tail groups of lipids than that of polar surfaces, thus reducing the mobility of the lipid, leading to local nucleation sites. This chain of events disrupts local line tensions, resulting in the formation of lamellar podia-like features. These trends are consistent with the experimental observations, and the MD simulations help rationalize important local structural features, e.g., lamellar podia-shaped features, of POPC assemblies on nonpolar surfaces. The presence of glycerol was shown to strengthen the formation and stability of bilayers due to the formation of cross-linked networks between layers, especially as they are dehydrated, suggesting that glycerol could act like a sugar would in the water replacement hypothesis.^29^ For the stacked bilayer configuration that most closely resembles the Langmuir–Blodgett and Langmuir–Schafer processes, we observed previously shown effects such as lubrication and layer inversion for the initial attachment. The starting configuration of the simulations was shown to play a role in the entrapment of glycerol locally to the surface. This entrapment formed parallel bilayers in the case of nonpolar surfaces, unlike those assembled through random starting configurations. The insights revealed from simulations have significant impacts on the construction of nano- and mesoscale lipid structures by design, which will benefit the construction of liposomal-based structures for drug delivery and engineering of vaccines and even protocells.

## Supporting Information Available

Supporting material includes videos of simulated dehydration, snapshots of stacked bilayer starting configurations, and polar surface analysis for all mechanistic figures; molecular dynamics snapshots during solvent evaporation on polar and nonpolar surfaces without glycerol, six-membrane bilayer configuration; molecular dynamics snapshots of ethanol and glycerol membrane interactions; lipid density profiles in the *z* direction (bilayer normal) and centered around the center of mass of the phosphate head group beads (PDF)
GlyMemMore (MPG)
GlyMemNonpolar (MPG)
MemMore (MPG)
MemNonpolar (MPG)
NoSurf (MPG)
RandGlyMore (MPG)
RandomMore (MPG)
RandomNonpolar (MPG)

## Supplementary Material

la2c00636_si_001.pdf

la2c00636_si_002.mpg

la2c00636_si_003.mpg

la2c00636_si_004.mpg

la2c00636_si_005.mpg

la2c00636_si_006.mpg

la2c00636_si_007.mpg

la2c00636_si_008.mpg

la2c00636_si_009.mpg
