# Supplementary material for: Impact of Surface Polarity on Lipid Assembly under Spatial Confinement
Source: Langmuir. 2022 Jun 7;38(24):7545–57. doi: 10.1021/acs.langmuir.2c00636 (PMC9219405; doi:10.1021/acs.langmuir.2c00636)
Supplement: Supplementary file 1 — la2c00636_si_001.pdf [file la2c00636_si_001.pdf]

Supporting Information

The impact of surface polarity on lipid assembly under spatial confinement

Bradley S. Harris[1], Yuqi Huang[2], Arpad Karsai[2], Wan-Chih Su[3], Pallavi D. Sambre[4], Atul N. Parikh[3], Gang-yu Liu[2], Roland Faller[1*]

[1] Department of Chemical Engineering, University of California, Davis, California, 95616, United States
[2] Department of Chemistry, University of California, Davis, California, 95616, United States
[3] Department of Biomedical Engineering, University of California, Davis, California, 95616, United State
[4] Department of Materials Science & Engineering, University of California, Davis, California 95616

* corresponding author: rfaller@ucdavis.edu

Contents

1. Lipid Assembly During solvent evaporation 3
2. Solvent Permeation 5
3. Density Profiles 7
4. Mechanisms associated with dehydration 9

1. Lipid Assembly During solvent evaporation

As described in the manuscript, we analyzed molecular dynamics snapshots of the drying process on polar (yellow) and non-polar (blue) surfaces. Here we present the snapshots corresponding to the stacked bilayer configurations without (Fig. S1) and with glycerol (Fig. S2) respectively.

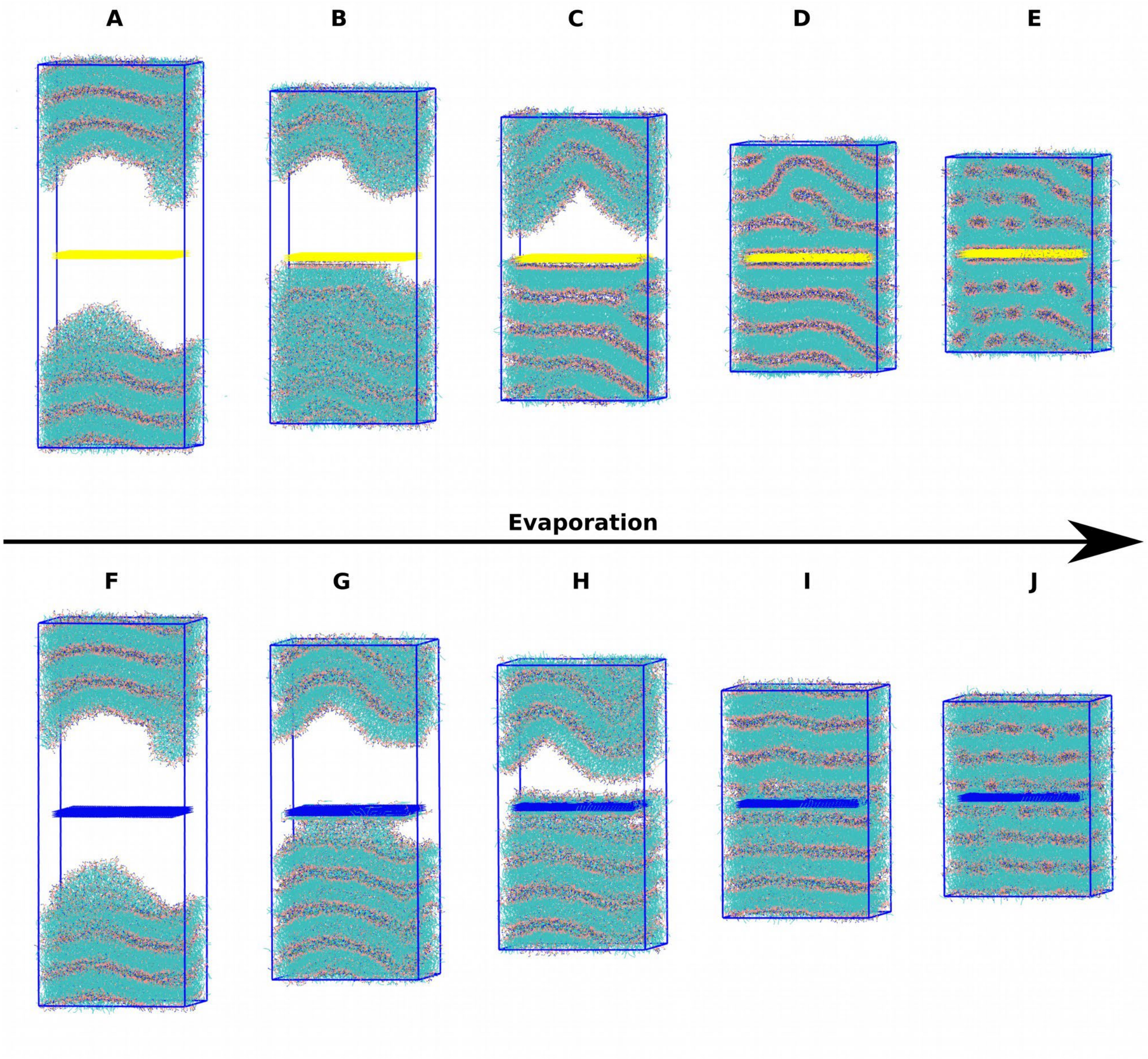

Figure S1: Molecular dynamics snapshots during solvent evaporation on polar and non-polar surfaces without glycerol, 6 membrane bilayer configuration. A) 100% solvent P4 surface. B) 75% solvent P4 surface. C) 50% solvent P4 surface. D) 20% solvent P4 surface. E) 0% solvent P4 surface. F) 100% solvent C1 surface. G) 75% solvent C1 surface. H) 50% solvent C1 surface. I) 20% solvent C1 surface. J) 0% solvent C1 surface.

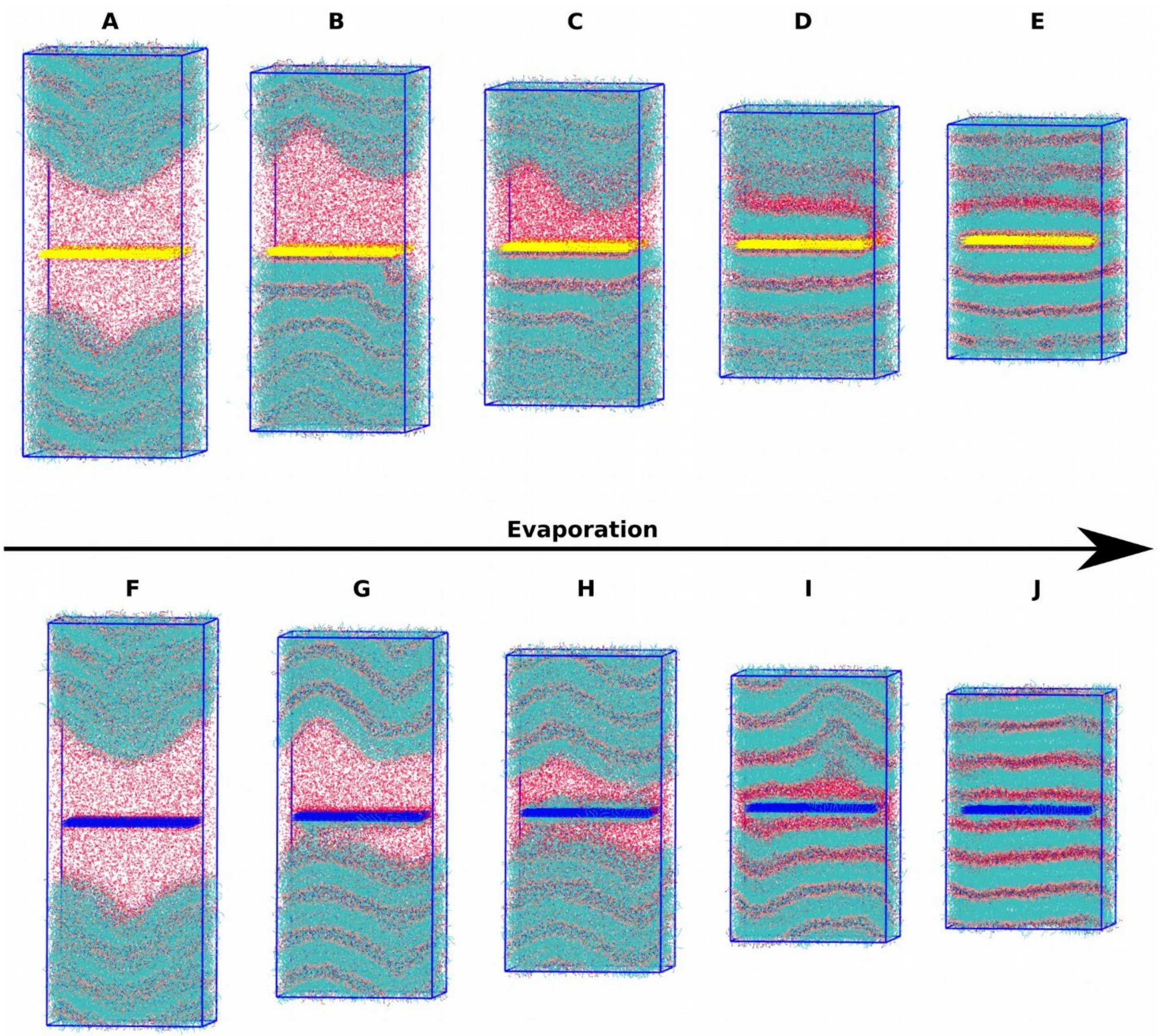

Figure S2: Molecular dynamics snapshots during solvent evaporation on polar and non-polar surfaces with glycerol, 6 membrane bilayer configuration. A) 100% solvent P4 surface. B) 75% solvent P4 surface. C) 50% solvent P4 surface. D) 20% solvent P4 surface. E) 0% solvent P4 surface. F) 100% solvent C1 surface. G) 75% solvent C1 surface. H) 50% solvent C1 surface. I) 20% solvent C1 surface. J) 0% solvent C1 surface

2. Solvent Permeation

As an extension to the analysis of membrane set up and structural effects during dehydration, snapshots of the ethanol and glycerol permeation and lipid surface distribution were taken. Figure S3 shows snapshots of initial membranes above a surface after 210 ns equilibration and before dehydration. S3A corresponds to single bilayer in pure ethanol, S3B-C to the 6 layer stack configuration with glycerol (shown in S2A), with B corresponding to the bottom bilayer closest to surface, and C to the bulk furthest from the surface. Coloring for the following figures corresponds as follows: Blue corresponds to carbonyl group in POPC, pink to glycerol backbone, yellow to phosphate, dark blue to NH3, red beads to glycerol solvent, silver beads to ethanol solvent.

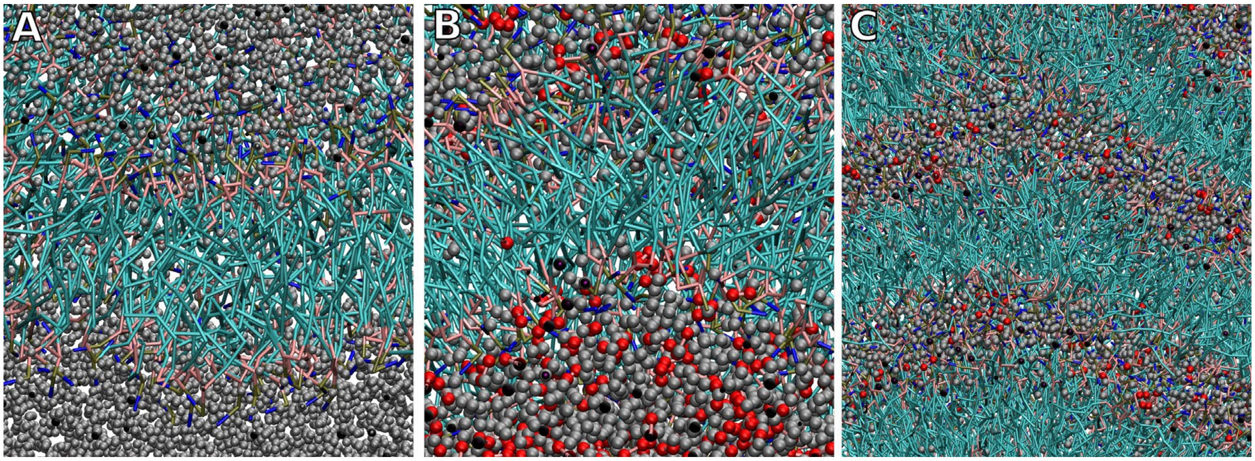

Figure S3: Molecular dynamics snapshots of ethanol and glycerol membrane interactions. A) Single membrane bilayer in pure ethanol. B) 6 bilayer stack in ethanol and glycerol, layer closest to surface. C) 6 bilayer stack in ethanol and glycerol, layers furthest from surface. Blue corresponds to carbonyl group in POPC, pink to glycerol backbone, yellow to phosphate, dark blue to NH3, red beads to glycerol solvent, silver beads to ethanol solvent.

To visualize how this changes during dehydration, similar snapshots were taken at the surface and in the bulk regions for the system on most polar surface with glycerol, again shown in Figure S2A-E. Figure S4 shows snapshots at 50%, 20%, and 0% initial ethanol remaining, with S4A-C corresponding to layers at the surface, and D-F representing layers furthest from the surface. Figure S5 shows the pore formed in figure S4E more clearly, by displaying membrane as lines instead of licorice.

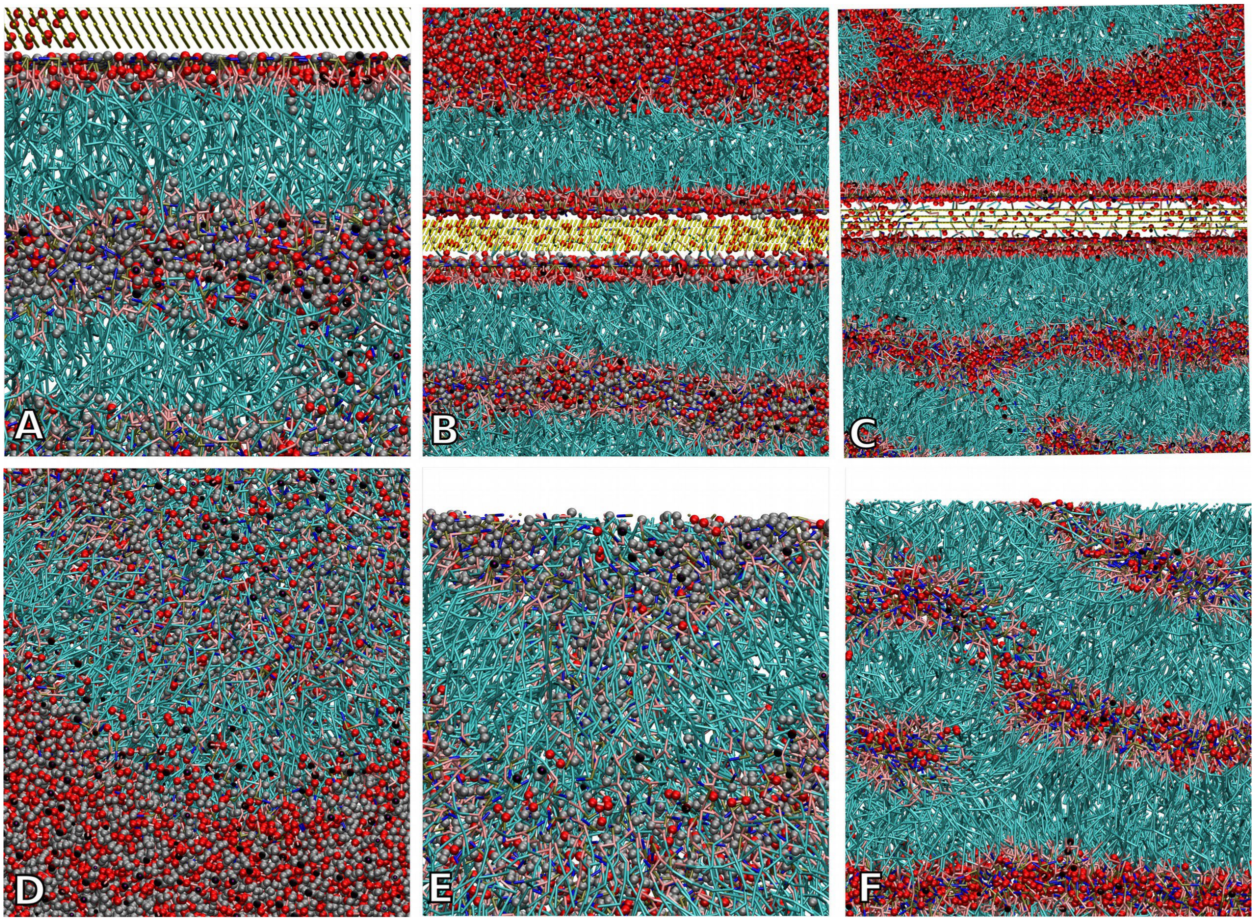

Figure S4: Molecular dynamics snapshots of ethanol and glycerol membrane interactions. 6 bilayer stack initial configuration with glycerol A) Layers closest to surface at 50% initial ethanol. B) Layers closest to surface at 20% initial ethanol C) Layers closest to the surface at 0% initial ethanol. D) Layers furthest from the surface at 50% initial ethanol E) Layers furthest from the surface at 20% initial ethanol F) Layers furthest from the surface at 0% initial ethanol. Blue corresponds to carbonyl group in POPC, pink to glycerol backbone, yellow to phosphate, dark blue to NH3, red beads to glycerol solvent, silver beads to ethanol solvent.

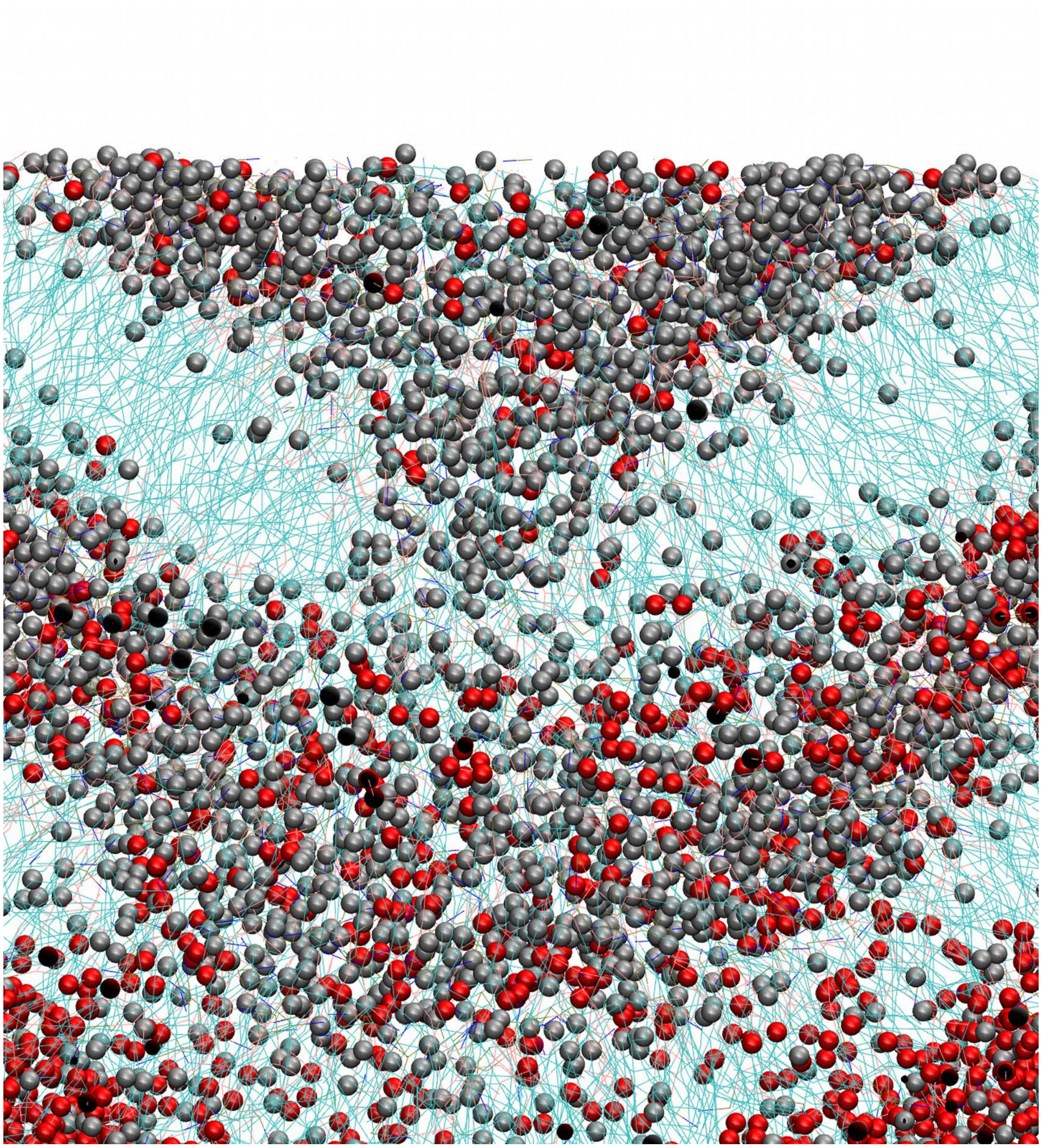

Figure S5: Molecular dynamics snapshots of ethanol and glycerol membrane interactions. Pore in Figure S4E displayed with lines instead of licorice.

3. Density Profiles

As described in the manuscript, density profiles were generated using the phosphate beads at 20% and 0% solvation with and without glycerol. The black lines correspond to very polar P4 surface, blue lines to non-polar C1 and red to the density profile of a stacked membrane away

from surface at full solvation with and without glycerol. The manuscript displays random starting configurations, here we show the 6 membrane stack configuration density profiles in Figure S6.

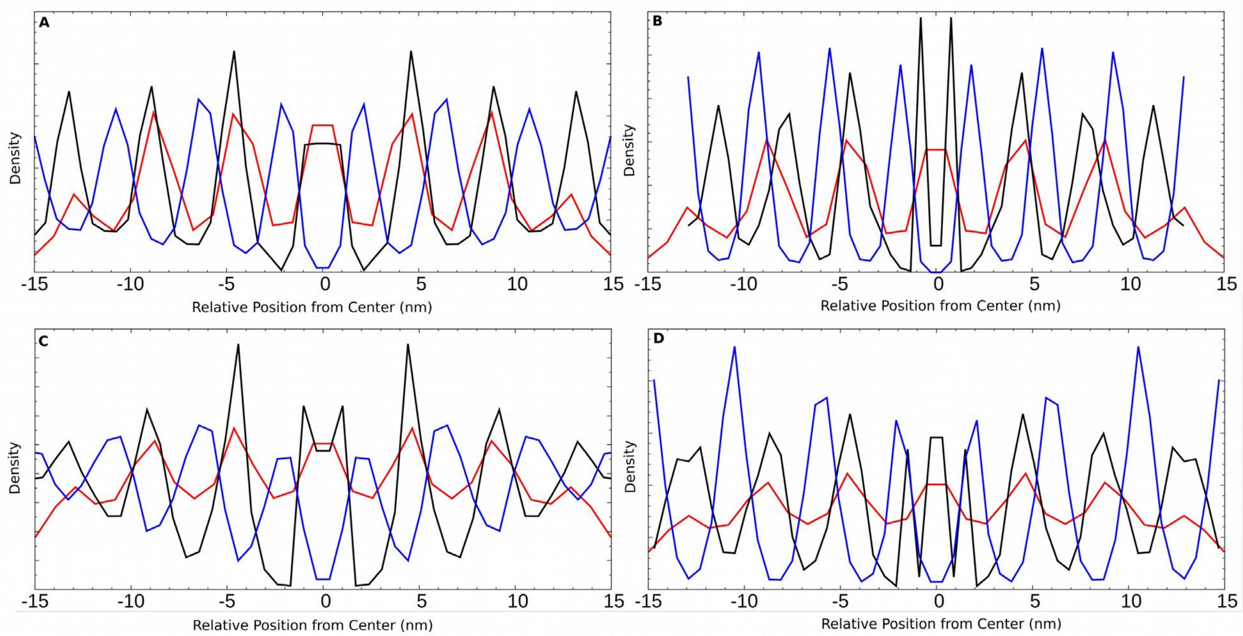

Figure S6: Lipid density profiles in the z direction (bilayer normal) and centered around the center of mass of the phosphate head group beads. A) No glycerol 20% of initial ethanol from bilayer start. B) No glycerol 0% of initial ethanol from bilayer start. C) 20% of initial ethanol with glycerol from bilayer start. D) 0% of initial ethanol with glycerol from bilayer start. Red line corresponds to control bilayer stack with or without glycerol, black lines to very polar P4 surface, blue lines to non-polar C1 surface.

4. Mechanisms associated with dehydration

As described in the manuscript, the potential energy, surface tension, and lipid tail order parameters were evaluated during evaporation. The manuscript only presented the results from C1 and P4 surfaces, here we present the results for all configurations tested including P1. These results are plotted as a function of percentage of remaining ethanol and shown in Figures S7 – S9.

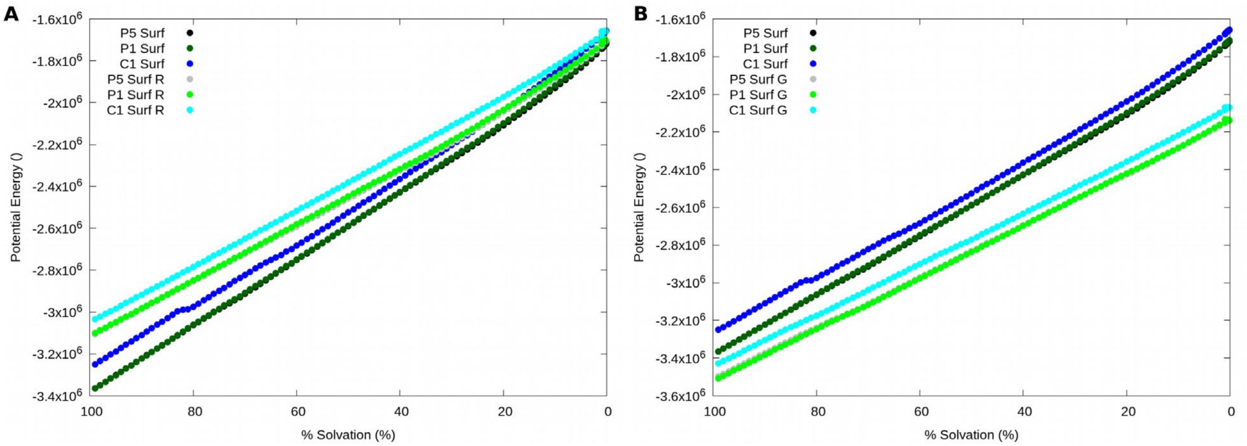

Figure S7: Potential Energies as a function of % of remaining initial ethanol. A) Bilayer stack vs random without glycerol. B) Bilayer stack without glycerol vs with glycerol.

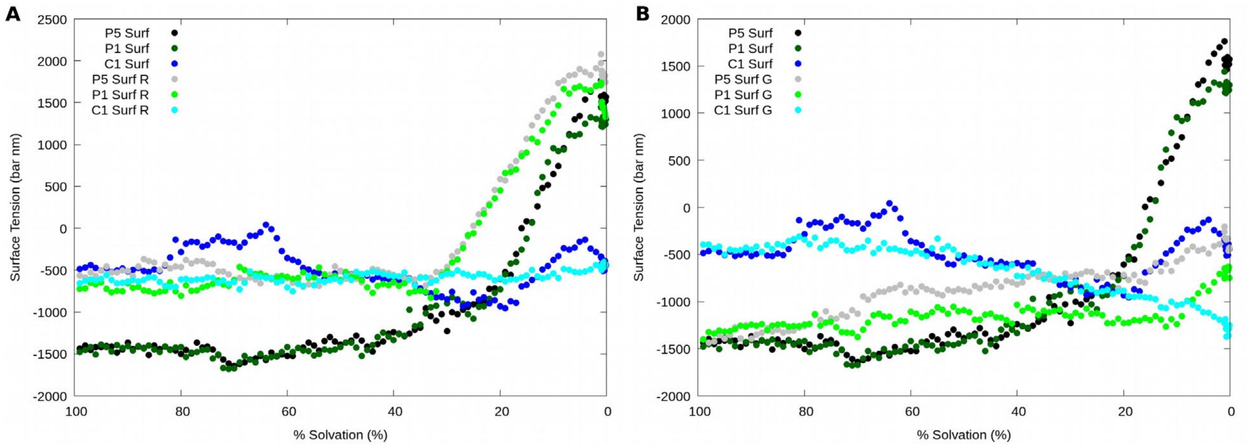

Figure S8: Surface tension as a function of % of remaining initial ethanol. A) Bilayer stack vs random without glycerol. B) Bilayer stack without glycerol vs with glycerol.

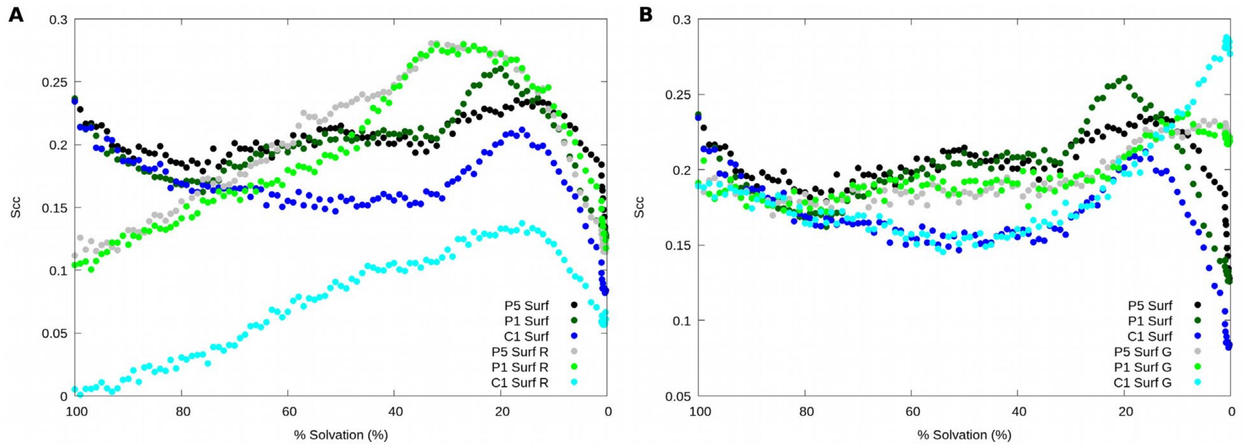

Figure S9: Tail order parameter as a function of % of remaining initial ethanol. A) Bilayer stack vs random without glycerol. B) Bilayer stack without glycerol vs with glycerol.
